# Pharmacological inhibition of MERTK induces in vivo retinal degeneration: a multimodal imaging ocular safety assessment

**DOI:** 10.1007/s00204-021-03197-8

**Published:** 2022-01-01

**Authors:** Gregory Hamm, Gareth Maglennon, Beth Williamson, Ruth Macdonald, Ann Doherty, Stewart Jones, Jayne Harris, James Blades, Alexander R. Harmer, Peter Barton, Philip B. Rawlins, Paul Smith, Jon Winter-Holt, Lindsay McMurray, Julia Johansson, Paul Fitzpatrick, William McCoull, Muireann Coen

**Affiliations:** 1 Clinical Pharmacology and Safety Sciences, R&D, AstraZeneca, Cambridge, UK; 2 Oncology R&D, AstraZeneca, Cambridge, UK; 3 Discovery Sciences, R&D, AstraZeneca, Cambridge, UK; 4 Clinical Pharmacology & Safety Sciences, R&D, AstraZeneca, Gothenburg, Sweden

**Keywords:** Retinal degeneration, MERTK, TAM kinases, Mass spectrometry imaging, Ocular histopathology, Ocular electron microscopy

## Abstract

The receptor tyrosine kinase, MERTK, plays an essential role in homeostasis of the retina via efferocytosis of shed outer nuclear segments of photoreceptors. The Royal College of Surgeons rat model of retinal degeneration has been linked to loss-of-function of MERTK, and together with the MERTK knock-out mouse, phenocopy retinitis pigmentosa in humans with MERTK mutations. Given recent efforts and interest in MERTK as a potential immuno-oncology target, development of a strategy to assess ocular safety at an early pre-clinical stage is critical. We have applied a state-of-the-art, multi-modal imaging platform to assess the in vivo effects of pharmacological inhibition of MERTK in mice. This involved the application of mass spectrometry imaging (MSI) to characterize the ocular spatial distribution of our highly selective MERTK inhibitor; AZ14145845, together with histopathology and transmission electron microscopy to characterize pathological and ultra-structural change in response to MERTK inhibition. In addition, we assessed the utility of a human retinal in vitro cell model to identify perturbation of phagocytosis post MERTK inhibition. We identified high localized total compound concentrations in the retinal pigment epithelium (RPE) and retinal lesions following 28 days of treatment with AZ14145845. These lesions were present in 4 of 8 treated animals, and were characterized by a thinning of the outer nuclear layer, loss of photoreceptors (PR) and accumulation of photoreceptor outer segments at the interface of the RPE and PRs. Furthermore, the lesions were very similar to that shown in the RCS rat and MERTK knock-out mouse, suggesting a MERTK-induced mechanism of PR cell death. This was further supported by the observation of reduced phagocytosis in the human retinal cell model following treatment with AZ14145845. Our study provides a viable, translational strategy to investigate the pre-clinical toxicity of MERTK inhibitors but is equally transferrable to novel chemotypes.

**Supplementary Information:**

The online version contains supplementary material available at 10.1007/s00204-021-03197-8.

## Introduction

MERTK is a receptor tyrosine kinase (RTKs) and part of the TAM family which also includes TYRO3 and AXL. The TAM family is involved in regulation of the innate immune response, for example, through MERTK-mediated efferocytosis of apoptotic cells by macrophages and of AXL-mediated dampening of TLR inflammatory responses (Graham et al. 2014). TAM RTKs are overexpressed in a wide variety of human cancers with their induction in tumor cells conferring a survival benefit. A body of evidence has arisen that inhibition of their kinase activity can be used as a strategy to reverse the immunosuppressive tumor microenvironment, and ultimately slow cancer cell proliferation (Davra et al. 2021; Peeters et al. 2020). Hence, there is increased interest in their potential as novel oncolytic therapeutic targets.

The key physiological role of MERTK lies in mediating phagocytosis of dying and dead cells, a process known as efferocytosis, which is performed by macrophages and to a lesser extent by monocytes, dendritic cells and epithelial cells (Boada-Romero et al. 2020). The retinal pigment epithelial (RPE) cells are the primary phagocytes of the eye, and mediate diurnal clearance of shed photoreceptor (PR) outer segments (POS). MERTK was identified as the causal gene in the Royal College of Surgeons (RCS) rat model of recessively inherited retinal degeneration (D'Cruz et al. 2000). A homozygous null mutation in MERTK in the RCS rat leads to the inability of the RPE cells to clear the shed POS. This results in accumulation of PR cellular debris, at the interface of the RPE cells and the PR outer layer and leads to post-natal PR cell death. Similarly, in mice that are homozygous for a targeted disruption of MERTK a retinal phenotype that is almost identical to the RCS rat is seen, with rapid, progressive degeneration of PRs (Duncan et al. 2003). As in the RCS rat, thinning of the outer nuclear layer (ONL) is seen from post-natal day 25 onwards with complete degeneration within 2–3 months consistent with almost complete loss of PRs and little detectable electroretinography (ERG) activity. Moreover, the ocular degeneration phenotype of both MERTK^−/−^ mice and the RCS rat mirrors that seen with human loss-of-function mutations of MERTK which result in retinitis pigmentosa, providing confidence in the translatability of pre-clinical findings and further evidence for the essentiality of MERTK in the retina (Gal et al. 2000).

Pharmacological evidence that inhibition of MERTK function can lead to ultra-structural changes in the retina was seen in a 14 day mouse study with the small molecule MERTK inhibitor; UNC-569 (Sayama et al. 2018). Inhibition of MERTK phosphorylation was observed in retinal tissue for up to 17-h post oral administration. No changes were detected by microscopic examination of standard haematoxylin and eosin (H&E)-stained tissue sections of the eye. Electron microscopic evaluation showed an accumulation of membrane-bound multi-lamellated bodies in the RPE cells, which the authors attribute to engulfed POS. In addition, increased chromatin condensation in the nuclei of the outer nuclear layer was suggestive of early apoptosis of PRs. The counter-intuitive increase in phagosomes and phagolysosomes is tentatively attributed to increased phagocytosis and may result from time of sampling during the non-peak period of disc shedding. Furthermore, the synchronization of MERTK phosphorylation, phagocytosis and circadian rhythms were explored in a follow-up 28-day mouse study with UNC-569 (Sayama et al. 2020). This showed that the severity of UNC-569 induced retinal toxicity was influenced by the time of dosing (dosing at 5.5-h and 22-h post light onset, Zeitgeber times (ZT))), which was associated with  the physiological MERTK phosphorylation window (ca. 2–5-h post light onset). The authors describe an increased severity in the ZT22 time-point (ER stress in RPE, apoptosis of PRs and reduced digestion of POS) whereas in the ZT5.5 group only reduced digestion of POS was seen.

Furthermore, MERTK-specific antibodies were explored as an immuno-oncology therapy and to test the hypothesis that differences in blood-retinal permeability may limit retinal exposure and subsequent adverse effects of antibodies (White et al. 2019). A 4-week cynomolgus monkey study was conducted, with multiple doses of 2 MERTK antibodies (30, 100 mg/kg, antibody injected IV on Days 1, 8, 15, 22, 29) and assessment of both histopathological and functional endpoints. No abnormal ophthalmic or ERG findings were detected; however, histopathological abnormalities were identified in all animals treated with the MERTK antibodies. Disruption of the integrity of the RPE was seen, with vacuolation of the outer segments of photoreceptors, displacement of RPE cells, and single cell necrosis of the outer nuclear layer.

Given the pre-clinical development of many selective MERTK inhibitors, together with late-stage development of kinase inhibitors that inhibit MERTK, a deeper mechanistic understanding of the impact of MERTK inhibition on retinal integrity is warranted. Furthermore, given the retina is terminally differentiated and hence degeneration of PRs is non-reversible, it is critical to assess the safety of MERTK inhibitors early in pre-clinical development and ultimately to identify tools that enable prediction of translational retinal toxicity. Given the timescale of degeneration in both the MERTK -/- mouse and RCS rat, we anticipated that it could take many weeks of dosing prior to manifestation of histopathological changes in the eye. The benefit of histopathological assessment was that we could capture much of the retina, given that different regions of the retina are prone to certain types of degeneration.

Mass spectrometry imaging (MSI) represents a novel molecular imaging technology that is applied to ex vivo tissue sections, to provide spatial, label free, simultaneous xenobiotic and endogenous molecular phenotypes (Brignole-Baudouin et al. 2012; Karlsson and Hanrieder 2017; Pareek et al. 2020; Swales et al. 2018). Several examples of MSI application to study the ocular distribution of drugs have been recently published (Boughton et al. 2020) for example, a PI3K/mTOR inhibitor (Liu et al. 2020), atropine (Mori et al. 2019) and chloroquine (Yamada et al. 2011). Spatially resolved molecular MSI data can be integrated with gold standard H&E tissue features generating “molecular histology” images (Walch et al. 2008). This has been successfully applied to identify unique distribution of lipids in discrete retinal layers including the RPE in a mouse model (Anderson et al. 2014) with a human tissue exemplar provided by Zemski-Berry et al. (2013).

We sought to apply a range of state-of-the-art technologies to assess the effect of MERTK inhibition on ocular integrity in a 28 day mouse safety study with a selective tool compound that had demonstrated efficacy in a range of pre-clinical xenograft and immuno-oncology models [e.g., MERTK Ba/F3 efficacy models, syngeneic MC38 model (McCoull et al. 2021)]. Firstly, we confirmed the presence and distribution of compound in the eye and retinal layers using mass spectrometry based multiscale imaging tools. Secondly, we characterized morphological changes which were attributed to exposure to our tool MERTK inhibitior in ocular tissue using histopathology, and ultra-structural changes with high-resolution transmission electron microscopy (TEM). Finally, we characterized the effect of our MERTK inhibitor on phagocytosis of POS in an in vitro human retinal cell model to assess back-translation from our in vivo study. The overarching strategy presented herein will prove of wider value in assessing the risk of ocular toxicity of novel compounds at a pre-clinical discovery stage.

## Materials and methods

### Potency assays

Biochemical assays for Mer, Axl, Tyro3 and Flt3 were performed using a Rapidfire LCMS method as previously described (Nissink et al. 2021). Cellular assays for pMer, pAxl, pTyro3, pFLT3 were performed in transiently transfected Cos-7 (Monkey: African green) cell lines as previously described. The macrophage efferocytosis assay was performed as previously described (Clark et al. 2019).

### Animals and dosing

All in vivo experimental procedures were conducted in accordance with United Kingdom legislation (Animals (Scientific Procedures) Act, 1986) and were compliant with the ARRIVE guidelines. 6–7-week-old female (20–25 g) C57BL/6 J mice (Charles River Laboratories Ltd, UK) were acclimatized to the facility for 10 days and then randomized to groups based on bodyweight; vehicle control (*n* = 6, animals 1–6) and MERTK inhibitor treatment (*n* = 9, animals 7–15). Animals were treated via oral gavage with vehicle control (60% v/v 0.1 M HCl, 20% v/v PEG 400, 20% v/v purified water pH 3.5–4.5) or AZ14145845 (200 mg/kg). All animals were dosed twice daily with a dose volume of 10 mL/kg for 28 days, with the final dose being administered on the morning of Day 29. The dose interval between the first and second dose on each day was 8 h. This dose was selected as it showed efficacy in a mouse Mer kinase Ba/F3 xenograft tumor efficacy model, provided continuous 24-h cover over MERTK (90-fold cover over MERTK Cos-7 cell IC_50_ at C_max_) and was well tolerated. One animal was taken off study early at Day 3 (animal 12) due to bodyweight loss and adverse clinical signs. Samples for histopathology were taken from 6 controls (animals 1–6), 8 treated animals (animals 7–11, 13–15) and for electron microscopy from 1 control (animal no. 1) and 3 treated animals (animals 8–10) and for MSI from 3 treated animals (animals 13–15).

### Plasma toxicokinetic analysis

A whole blood sample (32 µL) was collected via a tail vein prick into a K_2_-EDTA treated capillary tube, centrifuged at 1500*g* for 10 min at 4 °C, and plasma collected using a micropipette (non-EDTA Ref.no. 172292, Vitrex Medical A/S, Denmark). Samples were collected on Day 1 at 30 min, 2-h, 8-h (pre-second dose) and 24-h post the first dose. On days 7, 28, 29 samples were collected at 2-h post the first dose and at 8-h pre the second dose.

### Ocular exposure in the rat

Male pigmented Long Evans and non-pigmented Han Wistar rats were treated via intravenous infusion of AZ14145845 at a dose of 2 μmol/kg/h in a TEG:DMA:H_2_O (1:1:1) formulation, dose volume 4 mL/kg. At 4-h post the 15-min intravenous infusion, the animals were killed and the eyes extracted and homogenized to determine the concentration of AZ14145845 in the eye.

### Histopathological examination of fixed samples

Whole eyes were dissected at necropsy and immediately fixed in Davidson’s solution (methylated spirits 740P 30%, 40% formaldehyde v/v 20%, acetic acid 10%, distilled water 30% and 1% acid fuschin v/v (magenta indicator). Eyes (right side only) were routinely processed, embedded in paraffin wax and sectioned at 4 µM in a superior–inferior sagittal manner passing through the optic nerve head and including the anterior and posterior segments of the eye. A total of 7 sections per eye, collected at step intervals of 24 µM, were stained with haematoxylin and eosin, and examined by light microscopy by a pathologist blinded to the treatment group.

### Transmission electron microscopy (TEM)

Eyes from the left side were collected at necropsy. The retina was fixed in 2.5% glutaraldehyde in 0.1 M sodium cacodylate buffer (pH 7.4) at room temperature for 1-h followed by storage at + 4 °C. Following the primary fixation, the retina was rinsed with 0.1 M phosphate buffer and postfixed in 2% osmium tetroxide in 0.1 M phosphate buffer, pH 7.4 at 4 °C for 2-h. The retina was then stepwise ethanol dehydrated followed by stepwise acetone/LX-112 infiltration and finally embedded in LX-112 (Ladd). Semi- and ultra-thin sections were prepared using a EM UC 7 (Leica). The ultra-thin sections (approximately 60–80 nm) were contrasted with uranyl acetate followed by Reynolds lead citrate and examined in a HT7700 transmission electron microscope (Hitachi) operated at 100 kV. Digital images were acquired using a 2k × 2k Veleta CCD camera (Olympus Soft Imaging Solutions GmbH).

### Mass spectrometry imaging; sample preparation and analysis

Whole eyes were co-embedded in a Hydroxypropyl methylcellulose (HPMC, Sigma Aldrich, UK) and poly-vinylpyrrolidone (PVP, Sigma Aldrich, UK) hydrogel to enable time-efficient sectioning under comparable conditions for all specimens analyzed in an experiment (Dannhorn et al. 2020). Right eyes from 3 dosed animals were placed upright in peel-a-way moulds (Thermo Scientific, Waltham, Massachusetts, USA) pre-filled with ice cold embedding medium before snap freezing in dry ice-chilled isopropanol followed by a wash in dry ice-chilled isopentane to wash off the excess of isopropanol. The frozen moulds were kept on dry ice to allow evaporation of the adherent isopentane before sectioning. Whole eyes in block were then sectioned at 10 µm using a CM3050 cryo-microtome (Leica Biosystems, Nussloch, Germany) and thaw-mounted onto indium thin oxide (ITO) coated glass slides (Bruker Daltonics, Bremen, Germany) for matrix-assisted laser desorption/ionization (MALDI) MSI or onto Superfrost slides (Fisher Scientific, Loughborough, UK) for histological examination. Tissue section slides were stored at − 80 °C until analysis. Haematoxylin and eosin (H&E) staining was performed on adjacent eye sections and sections were imaged with Aperio CS2 digital pathology scanner (Aperio Tech, Oxford, UK), and visualized with ImageScope software (Aperio Tech.). For the quantitative MSI (QMSI) experiment, a stock solution of AZ14145845 was prepared at a concentration of 2000 μM in DMSO. Standards were prepared from the stock solution as 50, 40, 30, 20, 10 and 5 μM dilutions in 50/50 (v/v) methanol/water. Calibration spots for quantitation were spotted onto control eye sections on the day of analysis using a BioSpot Nanodispenser Workstation (BioFluidix GmbH, Freiburg, Germany). The Nanodispenser Workstation dispensed one 50 nL droplet of each standard. Due to size restrictions this resulted in two spots per section located on the posterior region of the eye. The spots were allowed to dry at room temperature before MSI acquisition. MSI analysis of tissue sections was carried out using a MALDI time-of-flight (TOF) (rapifleX, Bruker Daltonics) mass spectrometer. Whole slides were first coated with dihydroxybenzoic acid (DHB) MALDI matrix using the TM Sprayer (HTX Technologies, NC, USA) as previously described (Swales et al. 2018). Images were collected at 10 and 20 μm of lateral resolution in positive ion detection mode over a mass range of 300–1000 Da. FlexControl 4.0 and FlexImaging 5.0 (Bruker Daltonics, Bremen, Germany) were used for MS parameter optimization and MSI experimental set up, respectively.

Data management, analysis and visualization were performed using SCiLS Lab MVS 2020a software (Bruker Daltonics, Bremen, Germany). MS images were normalized to the total ion count (TIC) to compensate for signal instabilities. Unsupervised spatial clustering was applied on MSI data using a bisecting k-means algorithm with a weak pixel denoising and distance correlation as parameters using the 600–900 Da mass range. Line scan data were generated based on MSI analysis performed at 10 μm of spatial resolution. Regions of interest (ROIs) of 10 μm width (= 1 pixel) were set from the outer to the inner part of the back of the eye crossing all retinal layers (between 370 and 410 μm). Signal intensities of each compound were normalized against their maximum values (expressed in %) to have comparable data plotted against the ROI distance.

### Phagocytosis of photoreceptor outer segments (POS) in human retinal cells

ARPE19, human retinal epithelial cells, were cultured in Dulbecco’s modified Eagle’s medium (DMEM/F12, supplemented with 10% fetal bovine serum). All culture materials were purchased from Thermo Fisher Scientific. Cells were seeded at 10,000 cells per well in CellCarrier imaging plates (Perkin Elmer) and cultured for up to 6 weeks to allow for polarization of the cell layer. On the day of treatment cells were washed three times in PBS and then incubated in serum free conditions with FITC-labeled POS at a concentration of 10 POS/cell and either DMSO or 3 μM of AZ14145845 in DMSO. Following incubation for 6 h, cells were washed with PBS to remove non-internalized POS and fixed in 4% PFA. Following staining of nuclei with Hoechst, cells were imaged using a PerkinElmer ImageExpress Confocal microscope. Each well was imaged at 20 × with 16 images taken per well. Analysis modules were written using the custom module editor of MetaXpress vers 6.5.4.532. Nuclei were segmented and enumerated using the embedded count nuclei module. Individual POS particles were segmented and counted on a per image basis using the same custom module. Data are expressed as fold change in total POS per cell over control. Experiments were carried out a minimum of 3 times.

## Results

### Characterization of the MERTK inhibitor

As previously described, our novel imidazo[1,2-a]pyridine dual Mer/Axl type 1½ kinase inhibitor (AZ14145845, (McCoull et al. 2021)) displayed excellent on-target potency (MERTK pIC_50_ enzyme 9.0 and Cos-7 cell 7.7, Table 1) and activity in a macrophage efferocytosis assay (pIC_50_ 7.6). In addition, kinome profiling (Thermofisher coverage of 387 kinases at 1 μM) revealed excellent selectivity with > 90% inhibition of MERTK and AXL, and one additional kinase with > 75% inhibition: MAP4K5 (KHS1).

**Table 1:** Summary of the main physicochemical, pharmacokinetic and potency properties of AZ14145845 with corresponding molecular structure

Parameter	MERTK inhibitor
Structure	
Mer enzyme/cell (pIC_50_)	9.0/7.8
Axl enzyme/cell (pIC_50_)	7.9/7.0
Flt3 enzyme/cell (pIC_50_)	< 4.5/ < 4.5
Efferocytosis (pIC_50_)	7.6
hERG (µM)	5.7
LogD_7.4_	2.9
Solubility (µM)	938
Human/mouse PPB (% free)	14/7.6
Molecular weight	562
Mouse/rat hepatocyte clint (µL/min/ × 10^6^ cells)	14.8/21
HLM CL_int_ (µL/min/mg protein)	195
CaCo2 Papp (1e^−6^.cm/s)/efflux ratio	2.4/10

**Fig. 1:**
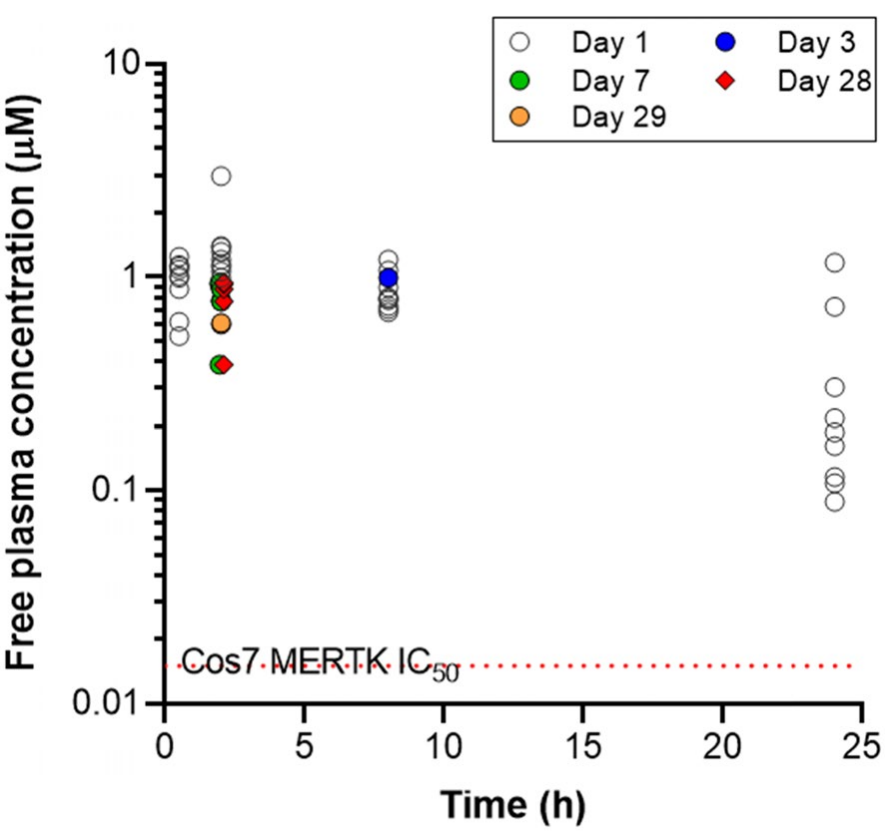
Free plasma concentration of AZ14145845 on day 1, 3, 7, 28 and 29 following 200 mg/kg BID dosing to C57BL/6 J mice

In vitro, AZ14145845 displayed encouraging ADME properties (Table 1); solubility was 938 µM, log*D*_pH7.4_ was 2.9 and mouse hepatocyte intrinsic clearance was 14.8 µL/min × 10^6^ cells. These data translated to acceptable pharmacokinetics in the mouse; clearance 44 mL/min/kg, volume of distribution at steady state 3.7 L/kg and bioavailability of 15%. Dose linear increase in C_max_ and AUC was observed for AZ14145845 up to 200 mg/kg. Following 200 mg/kg BID dosing to C57BL/6 J mice for 28 days, no accumulation was observed and the average free cover over MERTK Cos-7 cell IC_50_ was 90-fold at Cmax and 21-fold at Cmin (Fig. 1).

A minimal decrease in bodyweight was seen following treatment with AZ14145845 at day 2 (mean – 5.6% decrease from pre-dose day 1), day 3 (– 3.5% decrease from pre-dose day 1) and day 4 (– 1.6% decrease from pre-dose day 1). However, from day 5 onwards, animals had recovered and were above baseline weight and continued to gain weight in the region of 3–6% for the remainder of the study (SI Fig. 1).

To assist with compound characterization, the extent of melanin binding was assessed for AZ14145845 by comparing compound exposure in pigmented Long Evans rat and non-pigmented Han Wistar rats. The eye partition coefficient (Kp) in the pigmented rats was 19 compared to 2.5 in the non-pigmented animals. This ~ ten-fold difference in Kp was assumed to be a consequence of melanin binding as the eye microphysiology is expected to be consistent between strains of rat.

### Pathological examination

**Fig. 2:**
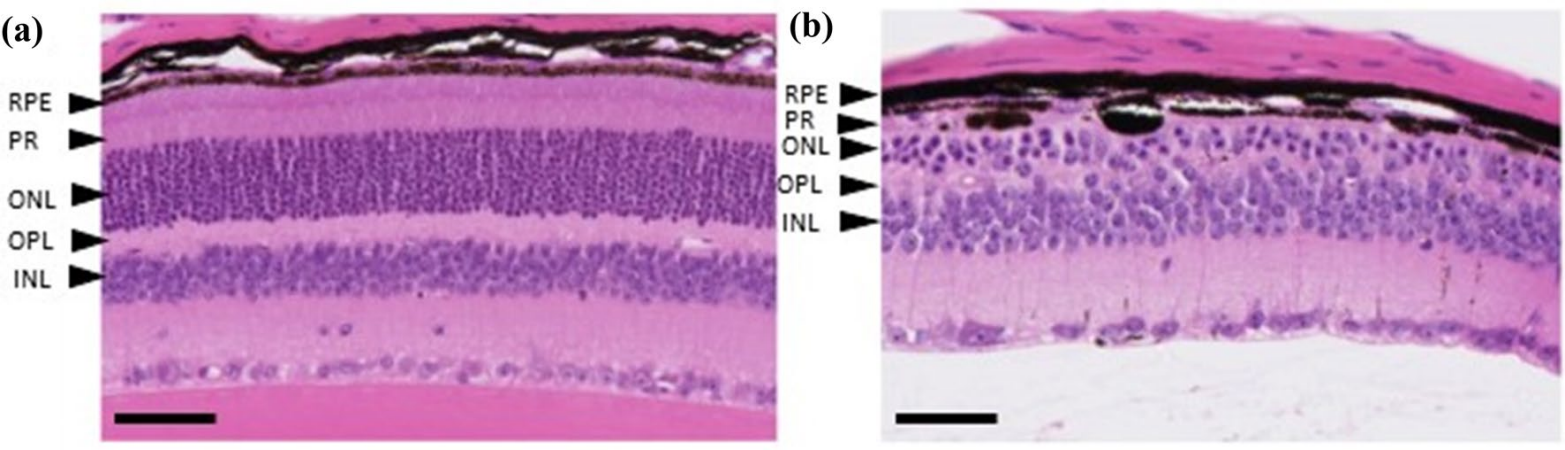
Retinal histopathology from FFPE H&E sections. **a** Animal no. 5 given vehicle only showing the normal layered structure of the retina, including Retinal Pigment Epithelium (RPE), Photoreceptors (PR), Outer Nuclear Layer (ONL), Outer Plexiform Layer (OPL) and Inner Nuclear Layer (INL). **b** Animal no. 10 given AZ14145845 shows marked thinning of the outer retina with loss of nuclei from the ONL and severe thinning of the ONL and PR layers and thinning of the PR containing displaced melanin pigment. Scale Bar = 50 µM

**Fig. 3:**
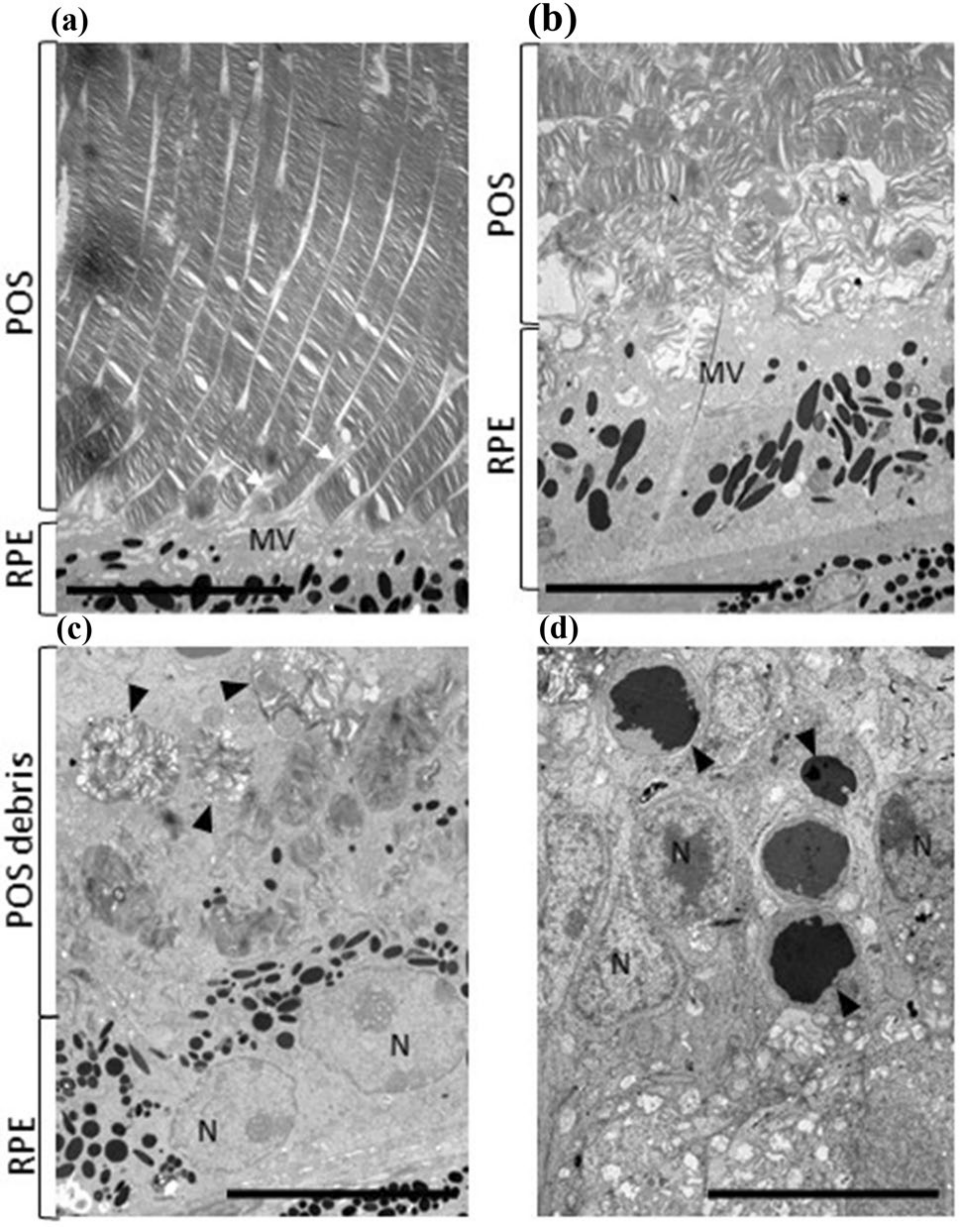
TEM micrographs: Ultrastructural changes in retinal layers by Electron Microscopy. **a** Transmission electron micrograph of interface between POS and RPE cells showing microvilli (MV) or RPE cells interdigitating (white arrows) with well-organised tips of POS in a vehicle-dosed animal. **b** Interface of RPE microvilli (MV) and POS in a AZ14145845-treated animal showing disorganization of POS and free strands of discs material (*). **c** Apical surface of RPE cells (*N* = nuclei) showing normally well-organised POS tips replaced by a zone of debris containing membrane-bound whorls (arrows) or POS material admixed with free degenerating POS material and microvilli. **d** Nuclei (N) of outer nuclear layer in a AZ14145845-treated animal showing several nuclei (arrows) with condensed chromatin. Scale bar = 10 µm

Light microscopic examination of H&E-stained sections from the right eye of each of the six mice given vehicle only and from four of the eight mice given AZ14145845 revealed no pathological changes (Fig. 2a and Table 2). Right eyes from the remaining four mice given AZ14145845 showed histopathology changes consistent with retinal atrophy (Animals No. 8, 9, 10 and 14). The outer layers of the retina were predominantly affected with marked atrophy characterized by loss of nuclei and subsequent thinning of the outer nuclear layer (ONL) and severe thinning of the outer plexiform and photoreceptor layers (Fig. 2b, Animal no. 10). Occasional nuclei of the ONL appeared condensed (pyknotic) compared to surrounding nuclei. In addition, there was flattening of the cells of the RPE and loss of apical microvilli. Animal numbers 8 and 9 had one or two focal lesions, respectively located towards the periphery of the retina and affecting approximately 20% of the length of the retina. Animal no. 10 had a focal lesion located midway between the optic nerve and the peripheral limit of the retina, affecting approximately 25% of its length. Animal no. 14 showed the most severe microscopic changes which affected the entire length of retina in the sections examined. TEM was performed on retina of the left eyes from one vehicle-dosed control animal (Animal no. 1) and from three animals dosed with AZ14145845 (Animals no. 8, 9 and 10, Fig. 3). All three MERTKi-treated animals showed focal lesions (retinal atrophy) on examination of H&E-stained sections by light microscopy in the contralateral right eyes. Semi-thin sections were stained with toluidine blue to select regions of interest for TEM. AZ14145845-treated animals showed ultra-structural changes at the interface between the POS and the RPE. In vehicle-dosed animals, normal POS consisted of membrane-bound, well-organized stacked layers of disc material and their tips were surrounded by apical microvilli of the RPE cells (Fig. 3a). In AZ14145845-treated animals, the normal interface was replaced by disorganized strands of disc material, some of which were membrane bound and some of which were not (Fig. 3b). The normal association between apical microvilli and POS was lost. In places, the interface between the POS and the RPE consisted of a disorganized debris zone consisting of RPE apical processes and fragmented photoreceptor discs (Fig. 3c). Moving away from the tips of the POS towards the inner segments, the normal organized appearance of the POS was typically retained. Occasional electron-dense nuclei with condensed chromatin were present in the outer nuclear layer (Fig. 3d).

**Table 2:** Retinal histopathology; incidence and severity in vehicle-treated mice (*n* = 6) and AZ14145845-treated mice (*n* = 8)

	Vehicle	Animal ID	MERTKi: AZ14145845	Animal ID
Number of animals with no H&E findings	6	Animals 1–6	4	Animals 7, 11, 13, 15
Minimal retinal degeneration/atrophy	0		3 with focal distribution	Animals 8, 9, 10
Moderate retinal degeneration/atrophy	0		1 with diffuse distribution	Animal 14
Total of any retinal degeneration/atrophy (of number examined)*	0 of 6		4 of 8	

*One eye per mouse examined

### AZ14145845 ocular distribution and endogenous molecular profile of the whole eye using MALDI-MSI

**Fig. 4:**
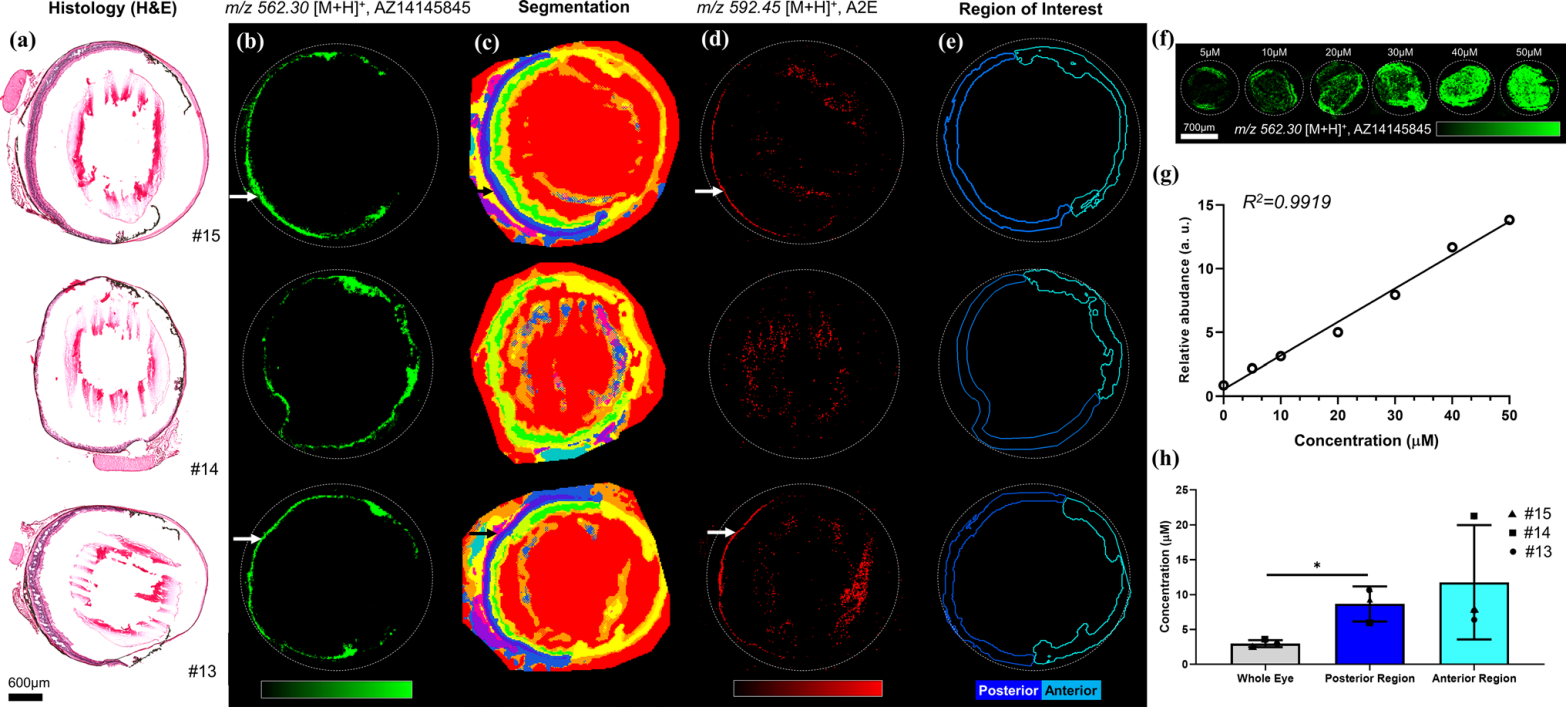
MALDI Mass Spectrometry Imaging analysis of whole eye sections treated with AZ14145845 obtained in positive ion mode at 20 µm spatial resolution. **a** Histological staining of adjacent eye sections using Haematoxylin and Eosin (H&E). **b** AZ14145845 molecular images from MSI. The white arrow shows the individual retinal layers. **c** Spatial segmentation. **d** A2E molecular images. **e** Region of interest describing the posterior and the anterior part of the eye used for the quantification of AZ14145845. **f** Molecular images of AZ14145845 dilution series spotted onto control eye section from 5 to 50 μM. **g** Calibration curve generated from AZ14145845 relative abundances on the dilution series molecular images. **h** Quantification of AZ14145845 in the whole eye, posterior and anterior segments in µm. Scale bar = 600 µm

MSI data obtained from whole eye sections showed the presence of AZ14145845, detected as a protonated ion, [M + H]^+^ at m/z 562.304 with a mass accuracy of 1.5 ppm, alongside equivalent H&E images from embedded mouse eye sections (Fig. 4a, Animals no. 13, 14, 15). AZ14145845 was distributed heterogeneously in the whole eye, and was largely localized in the pigmented retinal tissues of the RPE and choroid layers (Fig. 4b). AZ14145845 was also distributed in the iris and ciliary bodies of the anterior part of the eye. Segmentation maps generated from MSI analysis of three treated eyes cluster the spectra into groups of pixels based on a similarity index of molecular profiles (Fig. 4c; Alexandrov et al. 2010). These groups describe spatial features from the eye related to histology (detailed cluster tree provided in SI Fig. 2). Four main groups were identified based on the segmentation map corresponding to unique tissue features, #1 (red cluster) for vitreous and aqueous humors, #2 (orange clusters) for the iris, cornea and ciliary body, #3 (yellow/green clusters) inner and outer nuclear layers, #4 (blue/purple clusters) consisting mainly of the posterior part of the eye with RPE/choroid/sclera. These segmentation maps enable direct unsupervised comparison of the impact of AZ14145845 on the eye at the molecular level. Notably, animal no. 14 which had the most marked retinal lesion with a diffuse distribution showed the loss of cluster #4 compared to animals no. 13 and 15 (where no retinal lesion was detected by H&E). Furthermore, N-retinylidene-N-retinyl ethanolamine (A2E), which has been identified as an RPE regional molecular marker in several studies (Anderson et al. 2014), showed a dramatic decrease in animal no. 14 in the posterior part of the eye compared to the two other unaffected animals (SI Fig. 3). This retinoid molecule was identified from MSI data based on its exact mass at m/z 592.54 (SI Table 1), with only one pixel (20 µm) showing a high relative abundance of this ion in the retinal tissue (Fig. 4d). As the RPE is approximately 10 µm thick, it is challenging to discriminate this tissue from the choroid given the MSI data was obtained at 20 µm spatial resolution. Based on histology, spatial clustering and A2E distribution, we were able to delineate the posterior area (retinal tissue) from the anterior area of the eye and generate a so-called region of interest (ROI) for each section (Fig. 4e). These ROIs were used for total AZ14145845 quantification, where AZ14145845 was spotted onto a control eye section across a wide range of concentrations (Fig. 4f). A calibration curve was generated using TIC normalized mean relative abundance of AZ14145845 in the ROI of each calibration level (Fig. 4g). Thus, AZ14145845 was quantified based on its mean abundance in whole eye, posterior and anterior regions per animal by solving the linear regression function (details of quantification calculation are reported in SI Fig. 4). Total AZ14145845 concentration in the anterior (~ 11 µM) and posterior (~ 9 µM) regions are similar for all three animals, and significantly higher than in the whole eye (~ 3 µM), with a higher inter-animal variability seen in the anterior region (Fig. 4h).

**Fig. 5:**
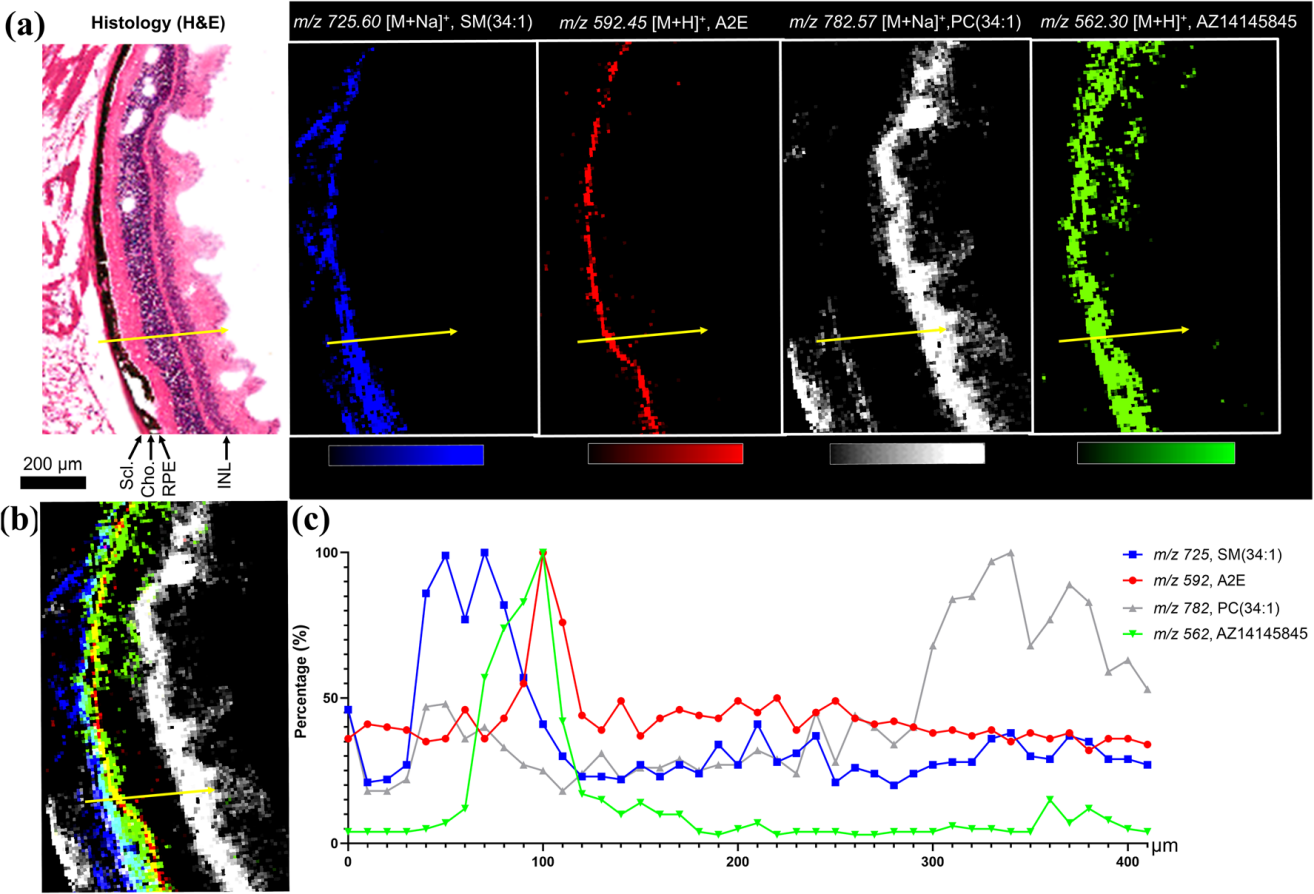
MALDI Mass Spectrometry Imaging of retinal layers of an AZ14145845-treated eye section (animal no. 13) at 10 µm spatial resolution showing co-localization of the MERTKi with the molecular marker of the RPE; A2E. **a** Histology of the eye with corresponding molecular images of Sphingomyelin, SM(34:1) at m/ 725, N-retinylidene-N-retinylethanolamine (A2E) at m/z 592, Phosphatidylcholine PC(34:1) at m/z 782 and MERTKi at m/z 562. **b** Overlay of molecular images and **c** line scan data from the region shown by the yellow arrow in (**a**)

High spatial resolution (10 µm) imaging data acquired on the posterior section of the eye (Animal no. 13) shows the spatial distribution of three molecular species and AZ14145845 together with a H&E-stained image from a serial section (Fig. 5a). A signal observed at *m/z 725.60* was identified as a sphingomyelin moiety, SM(34:1) which defined a part of the choroid and the outermost sclera region of the eye. As previously described A2E was used as an RPE histological marker. The inner nuclear layer (INL) together with inner plexiform/ganglion cell/nerve fiber layers of retinal tissues were highlighted by *m/z 782.57* identified using accurate mass as a phosphatidylcholine moiety, PC(34:1) (SI Table 1). Overlay distribution of the four ion images (Fig. 5b) together with line scan data show the relative abundance of the unique molecular species and AZ14145845 across the posterior section of the eye at a given point (Fig. 5c). Higher levels of AZ14145845 were found to be correlated with higher A2E values, additional line scans are reported in SI Fig. 5 and showed a similar correlation for AZ14145845 and A2E.

### POS phagocytosis in human retinal pigmented epithelial cells

**Fig. 6:**
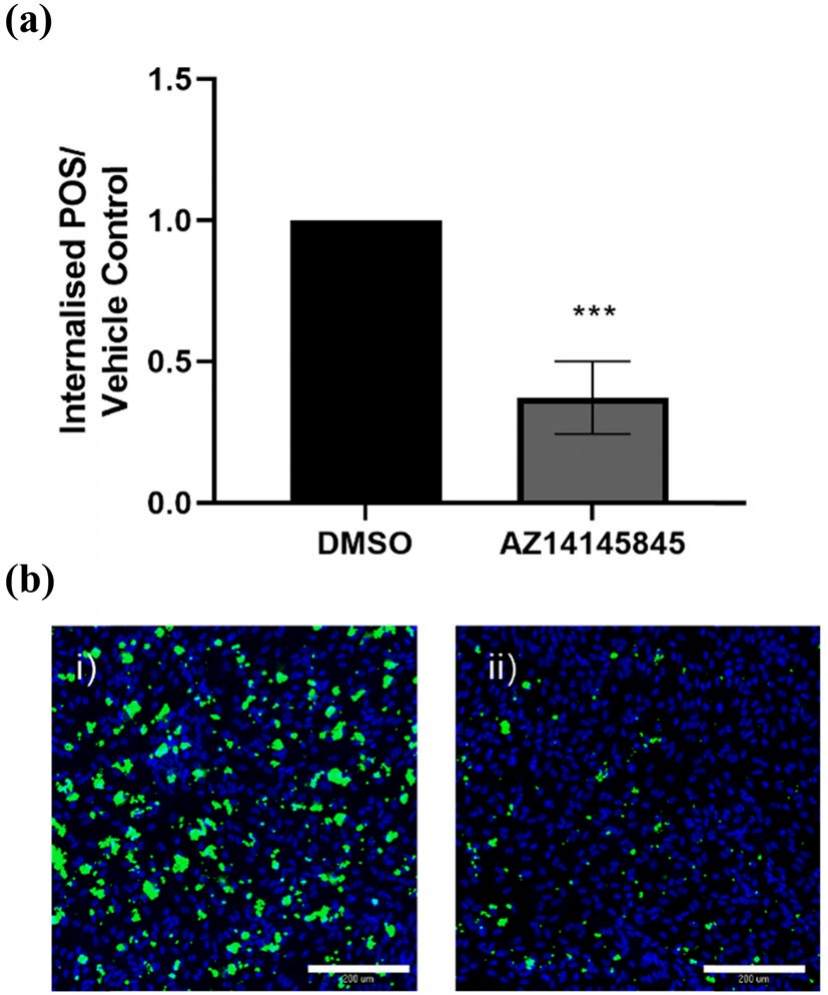
FITC-labeled photoreceptor outer segment phagocytosis in human retinal ARPE19 cells. **a** Quantification of total number of POS as determined by MetaExpress custom module for counting internalised POS particles expressed as fold change over vehicle control (mean ± standard deviation). Statistical significance was calculated using a one-way ANOVA (Graphpad Prism, ***p < 0.0001). **b** Representative images of (i) vehicle control cells and (ii) AZ14145845-treated cells. Nuclei are stained in blue and FITC-labeled POS are colored green. Scale bar = 200 µm

Following incubation with AZ14145845 for 6-h, a significant drop in the internalization of labeled POS was detected through fluorescent imaging (Fig. 6). Roughly, a 60% drop in the number of internalized POS were found in the compound treated wells indicating that AZ14145845 plays a role in blockage of the phagocytic mechanism involved in retinal epithelial clearance of POS.

## Discussion

We describe a pre-clinical strategy to investigate the potential for ocular toxicity of a novel and selective MERTKi; which involved the application and integration of state-of-the-art technologies including multi-modal MSI, histopathology and EM. This approach enabled in-depth characterization of an ocular lesion at both a molecular and morphological level. This strategy is equally applicable to characterize other small molecule inhibitors with a risk of ocular toxicity, which is of relevance given the increasing prevalence of clinical ocular toxicities particularly for tyrosine kinase inhibitors (Fu et al. 2017; Gokulgandhi et al. 2012). Photoreceptors are highly differentiated and lack an innate ability to regenerate, and together with the lack of robust circulating biomarkers of retinal toxicity, this highlights the critical need for retinal toxicity screening at an early discovery stage of drug development. Progress has been made with respect to characterization of the human miRNome (Karali et al. 2016), and identification of rodent panels of circulating miRNAs, such as the miR-183/96/182 cluster, which may have utility as biomarkers of retinal toxicity (Peng et al. 2016; Sayama et al. 2018).

The blood–retinal barrier (BRB) is comprised of both an inner and outer compartment, with expression of efflux transporters such as P-glycoprotein, multidrug resistance protein and breast cancer resistance protein on the inner BRB capillary endothelial cells, which serve to limit retinal drug exposure. In contrast to the blood–brain barrier, the expression levels of the efflux transporters on the BRB are lower resulting in higher ocular exposure for efflux substrates compared to brain exposure (Chen et al. 2013). Hence, understanding the relationship between efflux ratio and ocular exposure is crucial for PK understanding. As previously discussed (Williamson and Pilla Reddy 2021), melanin binding must be considered to accurately assess the free ocular exposure. However, differences in melanin levels across species and the localization of melanin within ocular tissues including the RPE layer (Jakubiak et al. 2018; Leblanc et al. 1998) hinders the interpretation of the free levels. Furthermore, a high degree of melanin binding may result in a prolonged elimination phase of the compound due to slow release from the protein (Robbie et al. 2013). Whilst melanin binding does not confer toxicity it will impact the observed efficacy and safety findings as defined by the free drug hypothesis. Hence, the comparison of the ocular Kp in pigmented and non-pigmented rats applied here, assisted with characterizing the extent of melanin binding.

This is the first report of retinal toxicity with a highly selective MERTKi which was detected by H&E light microscopic analysis, as previous reports with the small molecule MERTKi UNC-569 have identified retinal lesions with electron microscopic analysis only. Following 28 days of treatment with a selective MERTKi, we utilized MSI to provide evidence for high compound concentrations localized to the RPE. AZ14145845 was largely found in melanin containing tissue including the ciliary body, choroid and RPE. Quantitative MSI was used to measure the concentration of drug in the different eye compartments and showed that AZ14145845 was preferentially localized to the RPE layer. These data highlight the advantage of spatially resolved molecular imaging technology rather than analysis of entire organ homogenates by LC–MS, as the latter would have significantly under-estimated the concentration of AZ14145845 in the RPE.

Histopathological assessment identified marked ocular lesions with significant degeneration of photoreceptors, albeit with considerable inter-animal variation with both diffuse and focal lesions identified in 4 of 8 treated animals, and no identifiable change in 4 of 8 animals. Lesions in the retina of mice in this study subjected to pharmacological inhibition of MERTK are similar to those described in the RCS rat and MERTK -/- mice (D'Cruz et al. 2000; Duncan et al. 2003). At the histological level, mice in this study showed a loss of photoreceptors and thinning of the outer nuclear layer of the retina, similar to progressive thinning of the outer nuclear layer that has been described in both the RCS and MERTK-/- mouse. At the ultra-structural level, mice showed an accumulation of photoreceptor material at the interface with the RPE, similar to that shown in the RCS rat and MERTK-/- mice. A feature of MERTK KO mice is the persistence of pyknotic nuclei in the outer nuclear layer, presumed to be due to abnormal clearance of apoptotic nuclei, secondary to a defect in phagocytosis. Similarly, in this study, mice undergoing pharmacological inhibition of MERTK showed the presence of cell nuclei in the ONL containing condensed chromatin, consistent with pyknotic nuclei. The events leading to photoreceptor cell death in the RCS and MERTK-/- mice are thought to be secondary to a defect in phagocytosis of POS, leading to an accumulation of cellular debris and a consequent degeneration of photoreceptors. We demonstrated that AZ14145845 reduced POS uptake in the human retinal pigmented epithelial cell model, in support of this hypothesis. Hence, our results suggest that pharmacological inhibition of MERTK in the mouse can lead to a similar mechanistic sequence of events as in a genetic loss-of-function model, that ultimately manifest as retinal degenerative lesions, and that this would translate to human.

MSI data showed a loss of one spatial cluster identified as RPE/choroid layers together with a strong decrease of A2E for the animal showing the most severe histopathological lesions (#14). The modulation of this bisretinoid biosynthesized in RPE cells (Liu et al. 2000) is in agreement with the degeneration of photoreceptors observed by histology in the same animal.

We chose to use a pigmented strain of mice in our study; C57BL/6 as the seminal characterization of mouse MERTK -/- by histopathology and EM was carried out in this same strain (Duncan et al. 2003) and we wished to compare the effects of our MERTKi pharmacological inhibition with that of a genetic los-of-function model. In addition, the choice of a pigmented strain of mice most closely mimics the pigmented human eye, and hence has the greatest translational relevance. Furthermore, our syngeneic MC38 efficacy models also utilized this strain and hence, we had confidence in comparative target engagement across both efficacy and safety models (McCoull et al. 2021).

Rodents, including mice, can develop spontaneous retinal lesions including retinal degeneration which is typically age dependent; the incidence in pigmented female CD1 mice is 1.6% in studies of 4 weeks duration and increases to 5% in 2-year studies (Mukaratirwa et al. 2015). Hence, we would not expect background retinal lesions to confound our study given the young age of our mice, supported by a lack of detection of any retinal degenerative lesion in our control animals. Finally, background retinal lesions are more commonly detected in non-pigmented mice providing further justification of our rationale to choose a pigmented strain for our study.

One of the limitations of our study is that we lack direct evidence for target engagement in the eye and RPE; however, given the presence of high concentrations of our MERTKi in the RPE, the similarity of the observed microscopic change to the RCS rat model and mouse MERTK -/- model and translation to impaired phagocytosis in the in vitro human RPE model, we have confidence the effects we observe are due to direct ocular MERTK inhibition. In addition, our tool compound is well characterized and highly selective, and we identified no off-target risks from broad secondary pharmacology profiling, that could be linked to ocular toxicity and would confound interpretation of this work. Furthermore, we identified target engagement in a range of cell models and dose dependent in vivo efficacy and target engagement in MERTK-dependent mouse models (McCoull et al. 2021).

We are unable to explain the inter-animal variability with regard to the histological detection of a retinal lesion (4 of 8 animals) and would expect comparable target cover and ocular compound exposure based on the plasma pharmacokinetic profiles and MSI quantification of total MERTKi (3 animals). It is possible that had we dosed for longer than 28 days, we would have detected retinal lesions in all animals, and the temporal development of the lesion warrants further investigation. This possibility is supported by the RCS rat and MERTK -/-  mouse showing complete PR degeneration by 2–3 months of age. In addition, we selected eyes from animals with histological lesions to follow up with TEM (*n* = 3) and application of TEM to larger group sizes to detect any ultra-structural changes that preceded histological change would be merited going forward. We are also unable to explain the presence of focal versus diffuse lesions in our study, which may be linked to the above speculation on temporal discrepancies or due to differing susceptibilities of individual regions of the eye. It has been shown that a higher number of photoreceptor cells are located in the central region of the mouse retina (C57BL/6 J and BALB/c) and together with a thinner Bruch’s membrane than in the peripheral retina this would place a greater phagocytic load on the RPE (Volland et al. 2015).

RPE phagocytosis is regulated by circadian rhythms with the PR rod shedding initiating with the onset of light, with synchronized phosphorylation of MERTK and peak phagocytosis reported at 1.5–2-h post light onset in C57BL/6 mice (Mustafi et al. 2011) and at 3.5 h in BALBc mice (Sayama et al. 2018). We sampled eyes at 2-h post treatment which was approximately 4-h post light onset, which was rationalized as the most relevant sampling time in mouse given it covered the most likely window of peak phagocytosis. Given the association of time of dosing and target coverage with differential severity of retinal degeneration, characterized following treatment with UNC-569 (Sayama et al. 2020), it will be important in the future to fully explore the kinetics of retinal target engagement with diverse chemotypes.

Given the comparable expression of MERTK in the RPE of mouse, rat and human, together with the translation of a loss-of-function MERTK genotype to a retinitis pigmentosa clinical phenotype, the weight of evidence suggests significant utility of a mouse model in translation to the clinic. However, further work is required to understand the functional consequences of the retinal degeneration, using established techniques such as the electroretinogram; ERG.

## Conclusion

In conclusion, we have described a method to identify ocular toxicity and characterized the presence of our MERTK inhibitor within the ocular RPE. We have shown changes at the histological, molecular and ultra-structural levels by combining light microscopy, mass spectrometry imaging and electron microscopy to enable a holistic understanding of ocular compound exposure and subsequent pathological consequences. We also demonstrate translation of these in vivo effects to an in vitro human relevant RPE model, and hence taken together the ocular toxicity shown by AZ14145845 is likely to be as a consequence of MERTK inhibition and impaired clearance of POS. Consequently, there is a risk of ocular toxicity in humans and AZ14145845 was not considered suitable for further progression into clinical trials. The methods we have described will be useful in assessing other MERTKi at a discovery phase and prior to progression into clinical trials.

## Supplementary Information

Below is the link to the electronic supplementary material.

Supplementary file1 (DOCX 1626 KB)

## References

[CR1] Alexandrov T, Becker M, Deininger SO (2010). Spatial segmentation of imaging mass spectrometry data with edge-preserving image denoising and clustering. J Proteome Res.

[CR2] Anderson DG, Ablonczy Z, Koutalos Y (2014). High resolution MALDI imaging mass spectrometry of retinal tissue lipids. J Am Soc Mass Spectrom.

[CR3] Boada-Romero E, Martinez J, Heckmann BL, Green DR (2020). The clearance of dead cells by efferocytosis. Nat Rev Mol Cell Biol.

[CR4] Boughton BA, Thomas ORB, Demarais NJ, Trede D, Swearer SE, Grey AC (2020). Detection of small molecule concentration gradients in ocular tissues and humours. J Mass Spectrom.

[CR5] Brignole-Baudouin F, Desbenoit N, Hamm G (2012). A new safety concern for glaucoma treatment demonstrated by mass spectrometry imaging of benzalkonium chloride distribution in the eye, an experimental study in rabbits. PLoS ONE.

[CR6] Chen P, Chen H, Zang X (2013). Expression of efflux transporters in human ocular tissues. Drug Metab Dispos.

[CR7] Clark R, Usselmann L, Brown MR, Goeppert AU, Corrigan A (2019). A flexible high content imaging assay for profiling macrophage efferocytosis. J Immunol Methods.

[CR8] D'Cruz PM, Yasumura D, Weir J (2000). Mutation of the receptor tyrosine kinase gene MERTK in the retinal dystrophic RCS rat. Hum Mol Genet.

[CR9] Dannhorn A, Kazanc E, Ling S (2020). Universal sample preparation unlocking multimodal molecular tissue imaging. Anal Chem.

[CR10] Davra V, Kumar S, Geng K (2021). Axl and Mertk receptors cooperate to promote breast cancer progression by combined oncogenic signaling and evasion of host antitumor immunity. Can Res.

[CR11] Duncan JL, LaVail MM, Yasumura D, Yasumura D, Matthes MT (2003). An RCS-like retinal dystrophy phenotype in mer knockout mice. Invest Ophthalmol vis Sci.

[CR12] Fu C, Gombos DS, Lee J (2017). Ocular toxicities associated with targeted anticancer agents: an analysis of clinical data with management suggestions. Oncotarget.

[CR13] Gal A, Li Y, Thompson DA (2000). Mutations in MERTK, the human orthologue of the RCS rat retinal dystrophy gene, cause retinitis pigmentosa. Nat Genet.

[CR14] Gokulgandhi MR, Vadlapudi AD, Mitra AK (2012). Ocular toxicity from systemically administered xenobiotics. Expert Opin Drug Metab Toxicol.

[CR15] Graham DK, DeRyckere D, Davies KD, Earp HS (2014). The TAM family: phosphatidylserine sensing receptor tyrosine kinases gone awry in cancer. Nat Rev Cancer.

[CR16] Jakubiak P, Reutlinger M, Mattei P, Schuler F, Urtti A, Alvarez-Sánchez R (2018). Understanding molecular drivers of melanin binding to support rational design of small molecule ophthalmic drugs. J Med Chem.

[CR17] Karali M, Persico M, Mutarelli M (2016). High-resolution analysis of the human retina miRNome reveals isomiR variations and novel microRNAs. Nucleic Acids Res.

[CR18] Karlsson O, Hanrieder J (2017). Imaging mass spectrometry in drug development and toxicology. Arch Toxicol.

[CR19] Leblanc B, Jezequel S, Davies T, Hanton G, Taradach C (1998). Binding of drugs to eye melanin is not predictive of ocular toxicity. Regul Toxicol Pharmacol.

[CR20] Liu J, Itagaki Y, Ben-Shabat S, Nakanishi K, Sparrow JR (2000). The Biosynthesis of A2E, a fluorophore of aging retina, involves the formation of the precursor, A2-PE, in the photoreceptor outer segment membrane. J Biol Chem.

[CR21] Liu X, Liang X, LeCouter J (2020). Characterization of antineovascularization activity and ocular pharmacokinetics of phosphoinositide 3-kinase/mammalian target of rapamycin inhibitor GNE-947. Drug Metab Dispos.

[CR22] McCoull W, Boyd S, Brown MR (2021). Optimization of an Imidazo[1,2-a]pyridine series to afford highly selective type I1/2 dual Mer/Axl Kinase inhibitors with in vivo efficacy. J Med Chem.

[CR23] Mori N, Mochizuki T, Yamazaki F (2019). MALDI imaging mass spectrometry revealed atropine distribution in the ocular tissues and its transit from anterior to posterior regions in the whole-eye of rabbit after topical administration. PLoS ONE.

[CR24] Mukaratirwa S, Petterino C, Naylor SW, Bradley A (2015). Incidences and range of spontaneous lesions in the eye of Crl:CD-1(ICR)BR mice used in toxicity studies. Toxicol Pathol.

[CR25] Mustafi D, Kevany BM, Genoud C (2011). Defective photoreceptor phagocytosis in a mouse model of enhanced S-cone syndrome causes progressive retinal degeneration. FASEB.

[CR26] Nissink JWM, Bazzaz S, Blackett C (2021). Generating selective leads for Mer kinase inhibitors-example of a comprehensive lead-generation strategy. J Med Chem.

[CR27] Pareek V, Tian H, Winograd N, Benkovic SJ (2020). Metabolomics and mass spectrometry imaging reveal channeled de novo purine synthesis in cells. Science.

[CR28] Peeters MJW, Rahbech A, Thor Straten P (2020). TAM-ing T cells in the tumor microenvironment: implications for TAM receptor targeting. Cancer Immunol Immunother.

[CR29] Peng Q, Collette W, Giddabasappa A (2016). Editor's highlight: plasma miR-183/96/182 cluster and miR-124 are promising biomarkers of rat retinal toxicity. Toxicol Sci.

[CR30] Robbie SJ, Lundh von Leithner P, Ju M (2013). Assessing a novel depot delivery strategy for noninvasive administration of VEGF/PDGF RTK inhibitors for ocular neovascular disease. Invest Ophthalmol vis Sci.

[CR31] Sayama A, Okado K, Nakamura K (2018). UNC569-induced morphological changes in pigment epithelia and photoreceptor cells in the retina through MerTK inhibition in mice. Toxicol Pathol.

[CR32] Sayama A, Okado K, Yamaguchi M (2020). The impact of the timing of dosing on the severity of UNC569-induced ultrastructural changes in the mouse retina. Toxicol Pathol.

[CR33] Swales JG, Dexter A, Hamm G (2018). Quantitation of endogenous metabolites in mouse tumors using mass spectrometry imaging. Anal Chem.

[CR34] Volland S, Esteve-Rudd J, Hoo J, Yee C, Williams DS (2015). A comparison of some organizational characteristics of the mouse central retina and the human macula. PLoS ONE.

[CR35] Walch A, Rauser S, Deininger SO, Höfler H (2008). MALDI imaging mass spectrometry for direct tissue analysis: a new frontier for molecular histology. Histochem Cell Biol.

[CR36] White KF, Rausch M, Hua J, Walsh KF, Miller CE, Wells CC, Moodley D, Lee BH, Chappel SC, Holland PM, Hill JA (2019). MERTK-specific antibodies that have therapeutic antitumor activity in mice disrupt the integrity of the retinal pigmented epithelium in cynomolgus monkeys. Cancer Res.

[CR37] Williamson B, Pilla Reddy V (2021). Blood retinal barrier and ocular pharmacokinetics: considerations for the development of oncology drugs. Biopharm Drug Dispos.

[CR38] Yamada Y, Hidefumi K, Shion H, Oshikata M, Haramaki Y (2011). Distribution of chloroquine in ocular tissue of pigmented rat using matrix-assisted laser desorption/ionization imaging quadrupole time-of-flight tandem mass spectrometry. Rapid Commun Mass Spec.

[CR39] Zemski Berry KA, Gordon WC, Murphy RC, Bazan NG (2013). Spatial organization of lipids in the human retina and optic nerve by MALDI imaging mass spectrometry. J Lipid Res.

