# Supplementary material for: Pharmacological inhibition of MERTK induces in vivo retinal degeneration: a multimodal imaging ocular safety assessment
Source: Arch Toxicol. 2022 Jan 1;96(2):613–24. doi: 10.1007/s00204-021-03197-8 (PMC8837544; doi:10.1007/s00204-021-03197-8)

**Supplementary Information:**

*Pharmacological inhibition of MERTK induces in vivo retinal degeneration; a multimodal imaging ocular safety assessment.*

*Gregory Hamm^1¥^, Gareth Maglennon^1¥^, Beth Williamson^2^, Ruth Macdonald^1^, Ann Doherty^1^, Stewart Jones^1^, Jayne Harris^1^, James Blades^1^, Alexander R. Harmer^1^, Peter Barton^2^, Philip B. Rawlins^3^, Paul Smith^2^, Jon Winter-Holt^2^, Lindsay McMurray^2^, Julia Johansson^4^, Paul Fitzpatrick^4^, William McCoull^2^, Muireann Coen^1*^*

***¥Equally contributed to manuscript***

1. Clinical Pharmacology and Safety Sciences, R&D, AstraZeneca, Cambridge, UK
2. Oncology R&D, AstraZeneca, Cambridge, UK
3. Discovery Sciences, R&D, AstraZeneca, Cambridge, UK
4. Clinical Pharmacology & Safety Sciences, R&D, AstraZeneca, Gothenburg, Sweden

**Supplemental Table 1.** Assignments, cluster type and mass accuracy (in ppm) measurement for AZ14145845 and endogenous metabolites detected in eye by MSI (A2E, N-retinylidene-N-retinylethanolamine, SM, Sphingomyelin, PC, Phosphatidylcholine). Tissue specific detection of each molecular species is referenced in distinct retinal layers including NFL/GCL (nerve fibre layer/ganglion cell layer), INL (inner nuclear layer), retinal pigmented epithelium (RPE), choroid and sclera.

**Supplemental Figure 1**: Mean Bodyweight for control (group 1, blue) and AZ14145845 (group 2, red) across the 28 day safety study.


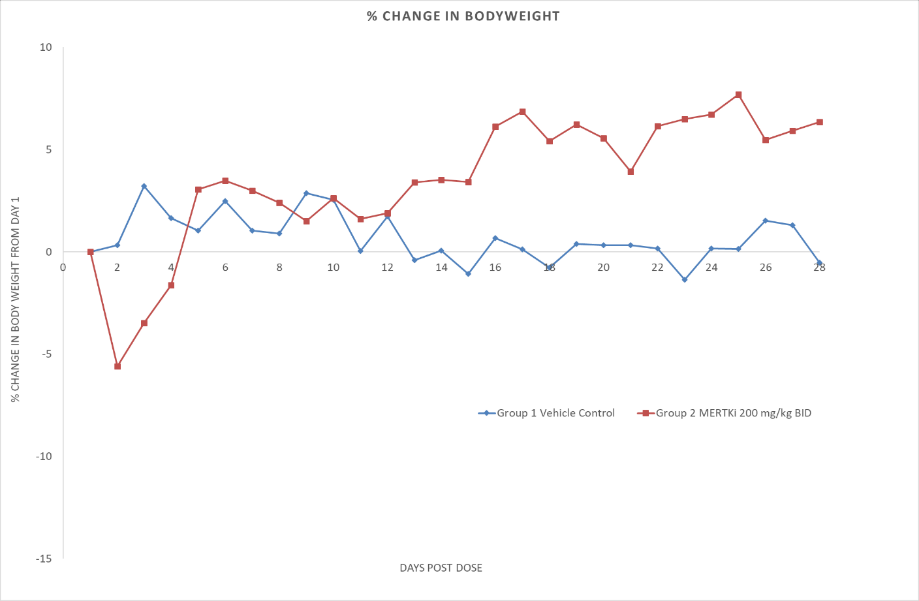
.

**Supplemental Figure 2.** Spatial segmentation of MSI data from an eye treated with AZ14145845. (a) Spatial segmentation map identified heterogeneous clustering phenotypes within the eye each of them with a specific colour; red (#1) for vitreous and aqueous humours, orange (#2) for the iris, cornea and ciliary body, yellow/green (#3) inner and outer nuclear layers, blue/purple (#4) consisting mainly of the posterior part of the eye with RPE/Choroid/Sclera. (b) H&E stain of the same eye sections for a direct comparison of the segmentation map with histology. (c) Cluster tree from k-means clustering representing the hierarchy and inter-connectedness of each cluster.


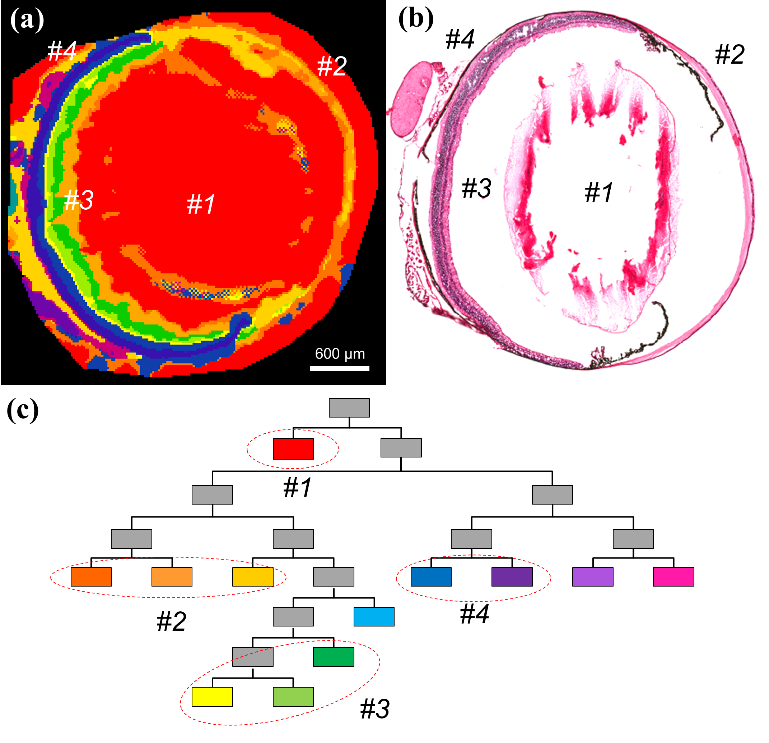


**Supplemental Figure 3.** Histogram of the mean relative abundance (in a.u. arbitrary units) of A2E (N-retinylidene-N-retinylethanolamine) in the posterior region of the eye from the three dosed animals, #15, #13 and #14 from figure 4.


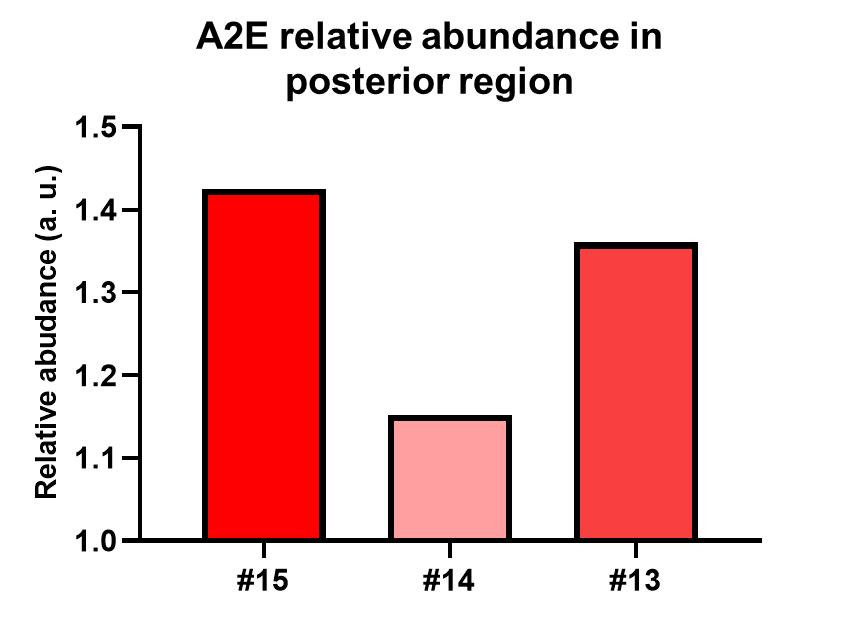


**Supplemental Figure 4.** Quantitative MSI data from the two technical replicates representing a **(a)** calibration curve with linear regression obtained from the dilution series of AZ14145845. **(b)** Histogram showing the mean concentration of AZ14145845 in whole eye as well as in the posterior and anterior segment of dosed animal #15, #14 and #13. **(c)** Statistics from the quantitative experiment including coefficient of regression (R^2^, function, limit of detection (LOD), normalization method used for MSI data i.e. Total Ion Count (TIC).


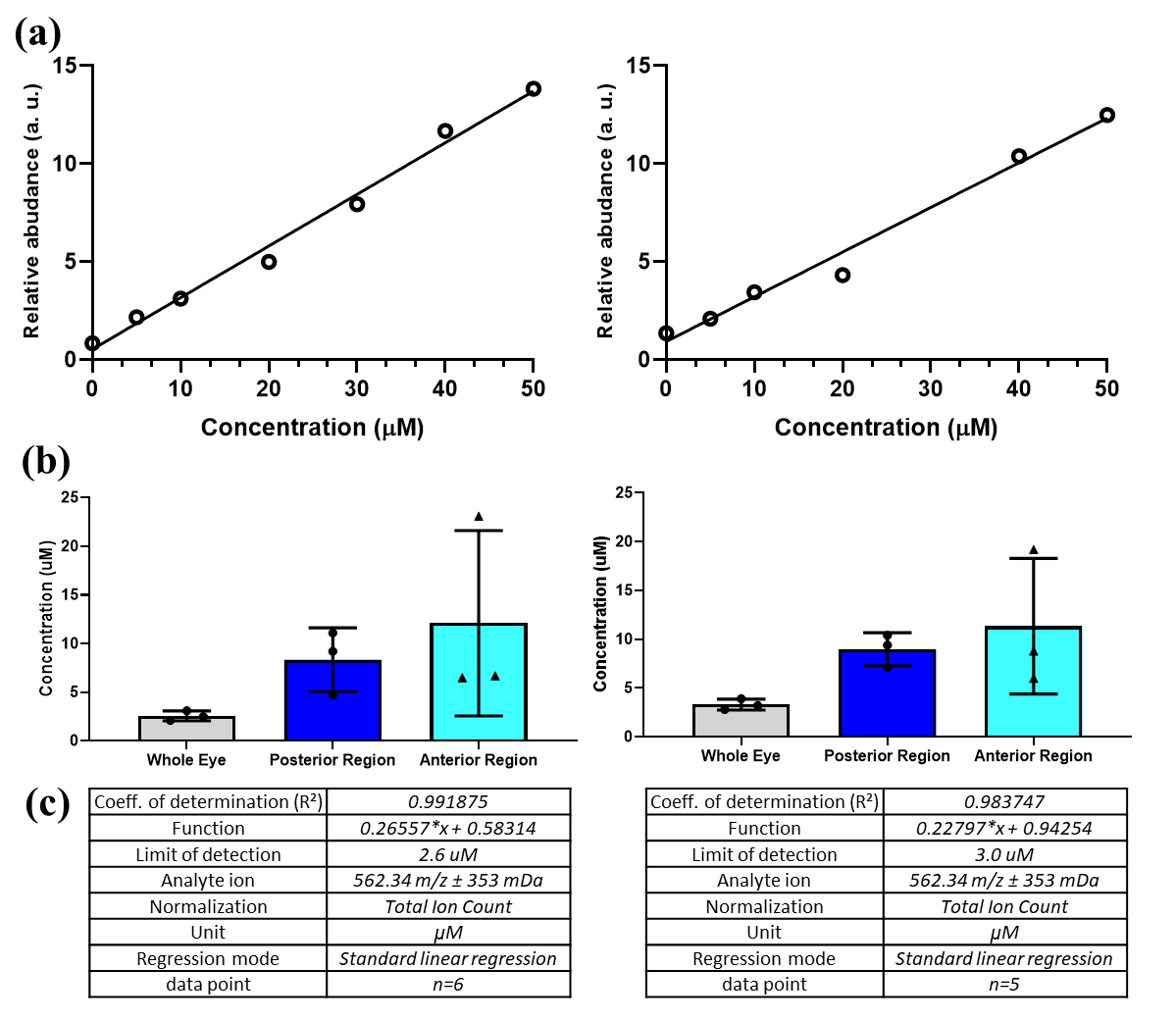


**Supplemental Figure 5.** MALDI MSI of retinal layers of AZ14145845 treated eye section (animal no. 13) at 10 µm of spatial resolution showing co-localisation of AZ14145845 with the molecular marker of the RPE (A2E). **(a)** Histology of the eye with **(b)** corresponding overlay molecular image of Sphingomyelin, SM(34:1) at *m/z 725*, *N*-retinylidene-*N*-retinylethanolamine (A2E) at *m/z 592*, phosphatidylcholine PC(34:1) at *m/z 782* and AZ14145845 at *m/z 562*. and **(c)** line scan data from 4 regions related to the 4 yellow arrows in **(a and b)**.


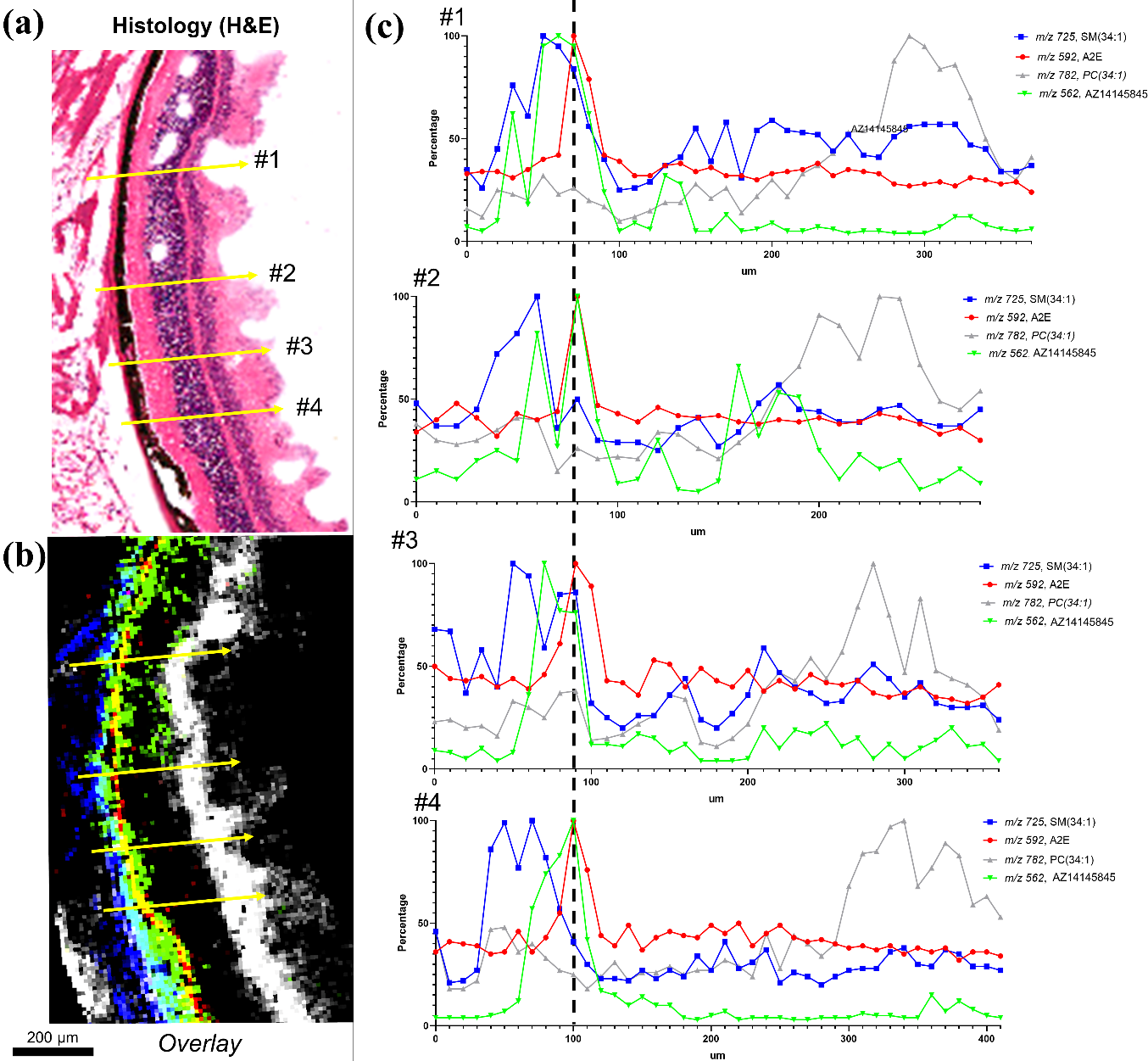

Supplement: Supplementary file 1 — Supplementary file1 (DOCX 1626 KB) [file 204_2021_3197_MOESM1_ESM.docx]
